# Recovering sedimentary ancient DNA of harmful dinoflagellates accumulated over the last 9000 years off Eastern Tasmania, Australia

**DOI:** 10.1093/ismeco/ycae098

**Published:** 2024-07-15

**Authors:** Linda Armbrecht, Christopher J S Bolch, Bradley Paine, Alan Cooper, Andrew McMinn, Craig Woodward, Gustaaf Hallegraeff

**Affiliations:** Institute for Marine and Antarctic Studies, University of Tasmania, Battery Point, TAS 7004, Australia; Australian Centre for Ancient DNA, School of Biological Sciences, Faculty of Sciences, The University of Adelaide, Adelaide, SA 5005, Australia; Institute for Marine and Antarctic Studies, University of Tasmania, Battery Point, TAS 7004, Australia; Institute for Marine and Antarctic Studies, University of Tasmania, Battery Point, TAS 7004, Australia; Gulbali Institute, Agriculture, Water and Environment, Charles Sturt University, Albury, NSW 2640, Australia; Institute for Marine and Antarctic Studies, University of Tasmania, Battery Point, TAS 7004, Australia; Australian Nuclear Science and Technology Organisation, Locked Bag 2001, Kirrawee DC, NSW 2232, Australia; Institute for Marine and Antarctic Studies, University of Tasmania, Battery Point, TAS 7004, Australia

**Keywords:** Alexandrium, Gymnodinium catenatum, Noctiluca scintillans, biotoxins, ballast water, seafloor

## Abstract

Harmful algal blooms (HABs) have had significant adverse impacts on the seafood industry along the Tasmanian east coast over the past 4 decades. To investigate the history of regional HABs, we performed analyses of sedimentary ancient DNA (*sed*aDNA) in coastal sediments up to ~9000 years old collected inshore and offshore of Maria Island, Tasmania. We used metagenomic shotgun sequencing and a hybridisation capture array (“HABbaits1”) to target three harmful dinoflagellate genera, *Alexandrium*, *Gymnodinium*, and *Noctiluca*. Bioinformatic and DNA damage analyses verified the authenticity of the *sed*aDNA sequences. Our results show that dinoflagellates of *Alexandrium* genera have been present off eastern Tasmania during the last ~8300 years, and we sporadically detected and unambiguously verified sequences of *Gymnodinium catenatum* that were present offshore up to ~7600 years ago. We also recovered *sed*aDNA of the fragile, soft-bodied *Noctiluca scintillans* with increased relative abundance since 2010, consistent with plankton surveys. This study enabled us to identify challenges of *sed*aDNA sequence validation (in particular for *G. catenatum*, a microreticulate gymnodinoid species) and provided guidance for the development of tools to monitor past and present HAB species and improvement of future HAB event predictions.

## Introduction

The negative impacts of harmful algal blooms (HABs) on tourism, aquaculture, fisheries, and human health mean that the appearance of novel HAB phenomena regularly raise the question of whether the species responsible is a recent introduction (e.g. via ballast water [1, 2]) or a previously cryptic endemic species stimulated by changing environmental conditions [3] or extreme climate events [4]). However, only a few studies have systematically examined the “cryptic species” hypothesis by investigating long-term dynamics over thousands of years (e.g. [5–7]).

The ocean environment off eastern Tasmania is a well-documented climate change hotspot characterised by a strengthening East Australian Current and rapidly increasing ocean temperatures (2.3°C increase since the 1940s [8]). The consequences of this oceanographic change are being detected in coastal marine communities, including changes in plankton composition and the appearance of previously unrecorded HAB species [9]. Three previously documented examples that are the focus of this study are the HAB dinoflagellates *Gymnodinium catenatum*, *Noctiluca scintillans*, and *Alexandrium* species [10, 11].

*G. catenatum* produces paralytic shellfish toxin (PST) and is the only toxic member of a phylogenetically distinct lineage within *Gymnodinium* (*G. catenatum*, *G. inusitatum*, *G. microreticulatum*, *G. nolleri*, and *G. trapeziforme*) that produce fossilising resting cysts with distinctive surface reticulation—referred to hereafter as “microreticulate species.” Thought to have been introduced to Tasmania in the 1970s by shipping ballast water [1, 2], *G. catenatum* first bloomed in the mid-1980s, causing PST contamination levels up to 250-fold above acceptable limits and extensive shellfish farm closures in the Derwent–Huon estuaries from 1986 to 1993 [12]. Supporting evidence for the recent introduction of *G. catenatum* includes detection of cysts in ship ballast tanks [13], lack of cysts in marine sediment dated prior to the early 1970s [2], reproductive compatibility studies [14], and molecular evidence from DNA fingerprinting [15] and rRNA gene sequencing [1], all of which support a link between Australasian populations and source populations from the Seto Inland Sea in southern Japan.

*N. scintillans* was first documented in Australia from Sydney Harbour [16], but since the 1990s has increasingly caused highly visible red tides and bioluminescence, resulting in frequent temporary closures of popular Sydney tourist beaches [17]. Amongst the suggested causes of *N. scintillans* blooms are eutrophication and coastal upwelling generating more diatom prey [18, 19]. *Noctilucascintillans *was first observed in Tasmanian waters in 1994 and are presumed to have dispersed southward with the East Australian Current, presenting a new threat to the salmonid fish farm industry from 2002 [11]. In 2010, *N. scintillans* was detected in the Southern Ocean for the first time, 240 km south of Tasmania, raising concerns of grazing impacts on iconic krill-based food webs [20].

Winter–spring blooms of the cool-temperate dinoflagellate *Alexandrium catenella* were recorded for the first time in 2012 and resulted in PST contamination levels up to 150 mg saxitoxin equivalents/kg, widespread seafood harvest closures, and public health warnings along Tasmania’s eastern coast [10, 21], and four non-fatal cases of human PST poisoning [22, 23]. The morphologically identical but genetically distinct *Alexandrium. australiense* and *Alexandrium pacificum* were previously known from Tasmanian waters [1]; however, *Alexandrium catenella* (= *Alexandrium tamarense* Group 1; = *Alexandrium fundyense*; [24]), had not previously been detected in Australasian waters. Analyses of regional populations indicated that Tasmanian *A. catenella* microsatellite DNA are divergent from those of other global populations [25], suggesting that *A. catenella* is either endemic or has dispersed to the area naturally over millennial timescales. Thus, *A. catenella* may be a previously cryptic population recently stimulated by changing environmental conditions, such as increased winter water column stratification [4].

Marine sediments are an archive of highly resistant sub-fossil resting cysts that represent the long-term dynamics of HAB species and can be used to address key questions about their introduction, disappearance, and reappearance in a region. Analysis of sedimentary ancient DNA (*sed*aDNA) also offers the distinct advantage of a record of non–cyst formers or less well-preserved cysts currently not accounted for in microfossil-based ecosystem reconstructions [26, 27]. For example, *Noctiluca* has not been shown to produce cysts but can be detected using *sed*aDNA [28]. However, *sed*aDNA is highly fragmented (typically <100 bp) and occurs at low concentrations, and because any one target species represents only a small fraction of the total DNA present, *sed*aDNA is prone to contamination with modern DNA. Therefore, bioinformatic assessment of DNA damage patterns typical of degraded ancient DNA is critical to authenticate the results [29]. Detection of target species can be improved by enrichment of target sequences by hybridisation capture with RNA probe arrays [30].

In this study, we combined metagenomic shotgun sequencing and hybridisation capture using a previously designed array (HABbaits1) [30] to enrich taxonomic marker genes of HAB species (18S rRNA, 28S rRNA, internal transcribed spacer [ITS], ribulose-bisphosphate carboxylase, and cytochrome c oxidase subunit 1) from *sed*aDNA in eastern Tasmanian marine sediments. To support an ecological interpretation of regional community change in Tasmanian waters, we investigated the presence and palaeo-historical patterns of *Alexandrium*, *G. catenatum*, and *N. scintillans* over the past ~9000 years.

## Materials and methods

### Sediment core collection and preparation

**Figure 1:**
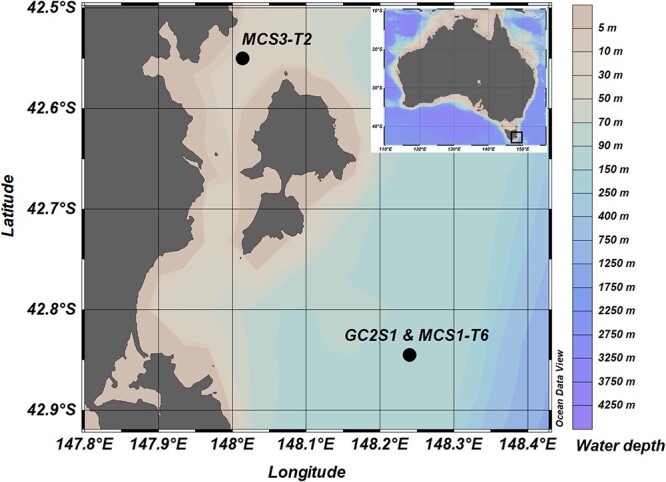
Sediment coring sites near Maria Island, Tasmania, Australia. Overview of coring locations offshore (gravity Core site 1, GC2S1, and multi-Core site 1 tube 6, MCS1-T6) and inshore Maria Island in the mercury passage (multi-Core site 3 tube 2, MCS3-T2). GC2S1 and MCS3-T6 were collected adjacent to each other and are depicted as one dot. Map created in ODV (Schlitzer, R., ocean data view, https://odv.awi.de, 2018).

An approximately 3 m–long sediment core (gravity core, designated “GC2S1”) and a parallel short core (12-cm multicore, designated “MCS1-T6”, where “T6” refers to tube 6 of the multi-corer) were collected in May 2018 during the RV (research vessel) *Investigator* voyage IN2018_T02 in 104-m water depth close to the continental shelf edge, east of Maria Island, Tasmania (Site 1; 148.240°E; 42.845^o^S) (Fig. 1). A 35 cm–long multi-core was also obtained from within Mercury Passage, west of Maria Island (Site 3; 42.550°S, 148.014°E) in a water depth of 68 m (designated “MCS3-T2”). All cores were immediately capped, sealed, labelled, and transported to the Australian Nuclear Science and Technology Organisation, Lucas Heights, Australia, where they were kept at 4°C. Cores GC2S1, MCS1-T6, and MCS3-T2 were opened, split, scanned (using a multi-function core scanning instrument [ITRAX] with X-ray fluorescence, radiographic X-ray, optical imaging, and magnetic susceptibility measurements), and subsampled for *sed*aDNA analyses in October 2018 (see [30] for details) using strict contamination control measures (facemask, hairnets, disposable coveralls, glove changes and surface/tool decontamination with 3% bleach and 70% ethanol between samples). Core GC2S1 was subsampled at 5-cm intervals; Core MCS1-T6 was subsampled at 2-cm intervals. Mercury Passage multicore (MCS3-T2) was subsampled at 2-cm intervals in the top 8 cm and then 5-cm intervals. All *sed*aDNA samples were immediately stored at −20°C. Hereafter, sediment depths refer to the top depths of each ~1.5- cm sample (diameter of sampling tube).

### Sediment dating

ITRAX scanning, X-ray fluorescence, radiographic X-ray, optical imaging, and magnetic susceptibility measurements confirmed excellent undisturbed preservation of cores. Age profiles were generated for MCS3-T2 and MCS1-T6 based on ^210^Pb measurements (8 and 6 dates, respectively); GC2S1 was based on both ^210^Pb (7 dates) and ^14^C (3 dates). A Bayesian age-depth model was constructed for each site using rbacon [31] on the R platform [32] with the SHCal20 curve for radiocarbon age calibration [33]. See Supplementary Material Note 1, Supplementary Material Fig. 1, and Supplementary Material Table 1 for details.

### *sed*aDNA extractions

Extractions of *sed*aDNA from 42 sediment samples and 7 extraction blank controls (EBCs) were carried out in ultraclean ancient (GC2S1) and forensic (MCS1-T6, MCS3-T2) facilities at Australian Centre for Ancient DNA, University of Adelaide, following ancient DNA decontamination standards [34]. We used the “combined” protocol [30, 35], which starts with incubation of a small subsample of 0.25 g of sediment in EDTA to isolate fragile eukaryote DNA [36], bead-beating to extract intracellular DNA from spores and cysts [28], and in-solution silica binding to target short DNA fragments (≥27 bp).

### Shotgun and hybridization capture sequencing library preparations

Metagenomic shotgun libraries were prepared from 20 μl of DNA extract using established techniques to create indexed, double-stranded libraries [30, 35, 37]. Magnetic beads were used to purify and size select DNA fragments under 500 bp [35]. The libraries were sequenced using Illumina NextSeq (2 × 75–bp cycle) at the Australian Cancer Research Foundation Cancer Genomics Facility & Centre for Cancer Biology (Adelaide, Australia), and the Garvan Institute of Medical Research, Kinghorn Centre for Clinical Genomics (Darlinghurst, Australia).

To maximise the *sed*aDNA yield from target dinoflagellates, hybridization capture with an RNA array (HABbaits1) targeting the harmful dinoflagellates in *Alexandrium* groups I–IV, *G. catenatum* and *N. scintillans* (see [30]). As input, we used at least ~ 50 ng of DNA in 7 μl, and 1 μl each of seven extraction blank controls in one “control-pool” of 7 μl. The hybridisation temperature was 65°C for the first 3 hours to favour highly specific binding, followed by a decrease to 60°C for another 37 hours of the hybridisation capture reaction. We prepared the beads by washing them twice with binding buffer and then adding binding buffer and 48 μl yeast tRNA (480 μg per 240 ml beads) in a third washing step, followed by brief vortexing and incubation of the solution on a rotary mixer (30 minutes, room temperature), pelleting on a magnetic rack, and two more washes with binding buffer. A final pool of 30 multiplexed sequencing libraries (2.75 nM) prepared from HABbaits1 (GC2S1 and MCS3-T2) were sequenced using Illumina HiSeq XTen (2 × 150– bp cycle) at the KCCG, Darlinghurst, Australia.

### Data analysis

Bioinformatic processing of sequence data was performed according to published protocols [35], using software and analytical parameters from Armbrecht et al. [30]. After filtering to remove low-complexity and duplicate reads, each dataset was processed without the standardisation step (i.e. without rarefying) to retain the maximum number of reads, which is crucial for ancient DNA damage analysis (see below). We also standardised the filtered shotgun data by subsampling (i.e. rarefying) to the lowest number of reads detected in a sample (2.2 million detected using seqtk v1.2), but this process resulted in significant reduction in the number of reads (Supplementary Material Note 2 and Supplementary Material Fig. 2). Please note that we used the words “reads” and “sequences” interchangeably throughout this study.

After quality control (FastQC v.0.11.4, MultiQC v1.8), the NCBI Nucleotide database (ftp://ftp.ncbi.nlm.nih.gov/blast/db/FASTA/nt.gz, downloaded November 2019) was used as the reference database to build a MALT index (Step 3) and sequences aligned using MALT (v0.4.0; semiglobal alignment) [38]. All resulting .blastn files were converted to .rma6 files using the Blast2RMA tool in MEGAN (v6_18_9, [39]). Subtractive filtering (i.e. subtracting reads for species identified in EBCs from samples) was conducted separately for the shotgun and HABbaits1 data [30]; however, no *Dinophyceae* taxa were detected in EBCs. For both datasets, read counts of all *Dinophyceae* nodes (from MEGAN6 v18.10) were exported for downstream analyses.

To assess *sed*aDNA damage, for our three target dinoflagellates, the “MALTExtract” and “Postprocessing” tools of the HOPS v0.33–2 pipeline [29] were run using the configurations of Armbrecht et al. [30]; e.g. taxalist *“b”*, including *Alexandrium* spp., *Gymnodinium* spp., and *N. scintillans* on shotgun and HABbaits1 *sed*aDNA data. The MALTExtract output, i.e. reads categorised as ancient (showing damage) or default (passing stringent filtering criteria but not showing damage) was exported and the proportion of *sed*aDNA damage per taxon determined. Damage profiles were generated using the MALTExtract Interactive Plotting Application (MEx-IPA, https://github.com/jfy133/MEx-IPA); however, due to low read numbers these graphics were not informative and were excluded from further analyses.

To test the reliability of short-sequence *sed*aDNA assignments, we created three dummy sample datasets, each containing sequences of one of the three dinoflagellate groups downloaded from NCBI (Supplementary Material Note 3). Each sequence was included with its complete length (Supplementary Material Table 2) and split into 56-bp fragments (corresponding to the average sequence length of our filtered shotgun data, see Results). If the last fragment was <25 bp it was excluded from the dummy sample (mimicking a minimum cutoff of 25 bp during sample data processing). Dummy samples were converted from .fasta to .fastq files (Supplementary Material Note 3) and processed via the same analytical pipeline used for samples.

Initial assessment of s*ed*aDNA assigned to *G. catenatum* indicated a significant proportion of sequences mapped to regions of the large subunit ribosomal RNA (LSU-rRNA) gene with limited database coverage of gymnodinoid dinoflagellates (Supplementary Material Note 4 and Supplementary Material Figs 4 and 5). Due to the potential for reference bias, we undertook additional sequencing of the D3 to D10 region (~1850 bp) of the LSU-rRNA of related microreticulate species available to us, *Gymnodinium microreticulatum* (strain CAWD191) and *G. nolleri* (strain K-0626) (Supplementary Material Note 4) and carried out detailed sequence validation. First, gymnodinoid-assigned reads were mapped to the *G. catenatum* reference sequence DQ785882, including comparative alignment with related *Gymnodinium* species (default alignment and assembly; Geneious Prime 2021). Direct match/mismatches to species in the alignment were used to determine the nearest-neighbour taxon and base-pair mismatches from the reference sequence for each read (Supplementary Material Fig. 5). Second, all *Gymnodinium* reads assigned to *G. catenatum* after the initial NCBI run were re-run against an in-house *Gymnodinium*-focused reference sequence database that included newly generated *G. microreticulatum* and *G. nolleri* LSU-rRNA D3–D10 sequences (Supplementary Material Note 5).

## Results

### Age model

The inshore core MCS3-T2 was dated to 77 years before 1950 at 34.5 cm below seafloor (cmbsf), i.e. the year 1873 (mean value). The offshore gravity core GC2S1 dated to 8878 years before 1950 at 268 cmbsf (~8945 years before sample collection). Comparing the ^210^Pb activity profiles from both GC2S1 and the adjacent multicore MCS1-T6, we estimated that ~3.5 cm were missing from the top of GC2S1, representing the last ~30 years (Supplementary Material Fig. 1).

### Validation of Alexandrium, Gymnodinium, and *Noctiluca scintillans* short-read species assignments

Dummy dataset analyses revealed that short *Alexandrium* spp. reads could only be reliably assigned to genus level (Supplementary Material Note 3, Supplementary Material Table 2, Supplementary Material Fig. 3). Thus, we interpreted this taxon at genus level. For *N. scintillans*, the majority of dummy sequences were correctly assigned and back-mapped to the NCBI sequence used to create our dummy sample (GQ380592.1) or another *N. scintillans* 18S rRNA reference sequence (AF022200.1). Rare mis-assignments of short 18S rRNA reads from *Alexandrium* were made to *Apicomplexa* and *Apocrita*, and to *Symbiodinium* and *Apocrita* for *N. scintillans*. For *G. catenatum*, most dummy reads were correctly assigned, but a few 18S rRNA reads were erroneously assigned to the apicomplexan *Eimeria*, *Streptophytina*, *Membracoidea*, or dinoflagellates *Durinskia baltica*, *Symbiodinium* sp. AW-2009, and microreticulate relative *G. microreticulatum*, suggesting *Gymnodinium* species assignment of short 18S rRNA reads (<56 bp) should be interpreted with caution. In most cases assignment uncertainty could be resolved by further inspection and verification (Supplementary Material Notes 4 and, 5, Supplementary Material Figs 4 and 5, Supplementary Material Tables 3 and 4).

**Figure 2:**
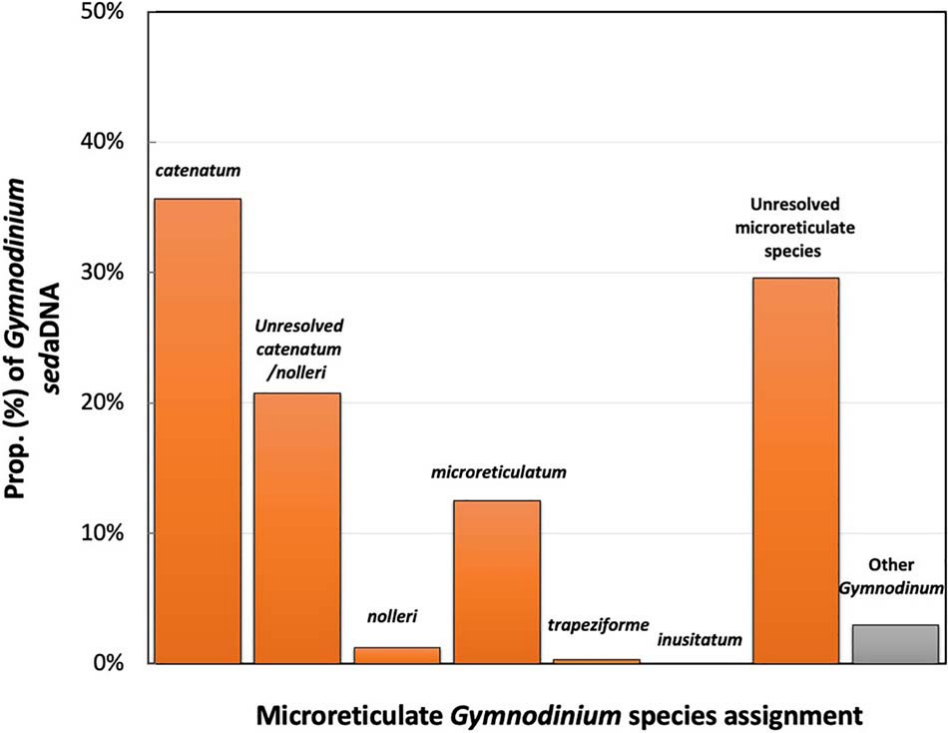
Proportion of *Gymnodinium sed*aDNA fragments assigned to each microreticulate *Gymnodinium* species after fragment mapping to a reference alignments (see Supplementary Material Note 5 for details). Unresolved categories represent *sed*aDNA mapping to gene locations/regions with insufficient sequence variation for unambiguous assignment.

Of a total of 352 HABbaits1 reads assigned to *Gymnodinium* from core GC2S1, 99% mapped to rRNA genes (Supplementary Material Note 4, Supplementary Material Fig. 5) and 97% were unambiguously assigned to microreticulate *Gymnodinium* species. More than half (56%) of the reads were closest to *G. catenatum*, with 36% unambiguously assigned to *G. catenatum*, 21% to rRNA regions in which we were unable to resolve *G. catenatum* from the closest relative *G. nolleri*, and 30% to regions with insufficient sequence variation to distinguish among microreticulate species (Fig. 2).

Re-assignment of *Gymnnodinium* reads using our in-house gymnodinoid-supplemented database improved microreticulate species resolution but resulted in more conservative species-level assignments than fragment mapping. Only 76% of reads could be assigned at the genus level, 28% to *G. nolleri*, 6% to *G. microreticulatum*, and 4% to *G. catenatum* (Supplementary Material Note 5, Supplementary Material Table 4).

### Representation of *Dinophyceae* in shotgun data

**Figure 3:**
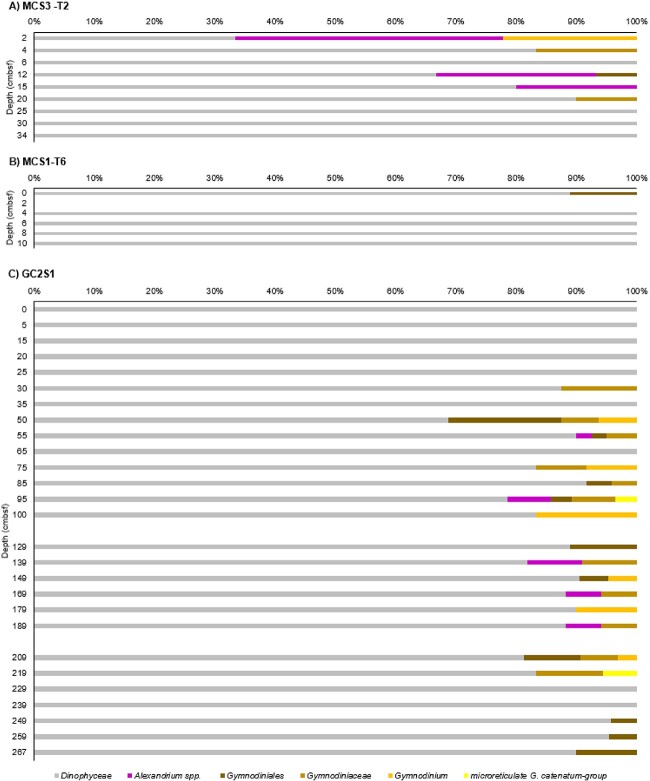
Relative *Dinophyceae* abundance (shotgun). *Dinophyceae* detected in non-rarefied shotgun data at Maria Island coring sites (A) MCS3-T2, (B) MCS1-T6, and (C) GC2S1. All non-target taxa are grouped as *Dinophyceae*, while *Alexandrium* and *Gymnodiniale*s and its subgroups are shown separately. Identity of *G. catenatum* should be interpreted as representing all microreticulate *Gymnodinium* species, and the genus *Alexandrium* (see Section 3.2). *N. scintillans* was not detected in the shotgun data. Total read count (all sites): 529.

From 42 shotgun samples we retrieved 824 503 filtered sequences across the three domains (*Bacteria*, *Archaea*, and *Eukaryota*), with 149 892 assigned to *Eukaryota* (18%). A total of 529 reads were assigned to *Dinophyceae* (0.06% of the domains and 0.35% of all eukaryotes). Harmful dinoflagellate sequences were detected in low abundance (19 *Alexandrium*, 13 *Gymnodinium*, and no *Noctiluca* sequences; Fig. 3). *Alexandrium* spp. were detected primarily in the upper 15 cmbsf at MCS3-T2 (last ~39 years) with few reads between 55 and 189 cmbsf at GC2S1 (~2600 and ~7200 years ago, respectively). Based on *Alexandrium* dummy data verification (see above) we conservatively assigned only to the genus level (Fig. 3). Three shotgun sequences were assigned to the microreticulate *Gymnodinium* group in GC2S1 at 95 cmbsf (~4400 years ago), and 219 cmbsf (~7800 years ago) (Fig. 3C). Microreticulate gymnodinoids were not detected in younger sediments of GC2S1 but were present in the more recently deposited surface sediments of the shorter MCS1-T6 core (Fig. 3B).

### Representation of *Dinophyceae* using HABbaits1

Hybridisation capture with the HABbaits1 array resulted in 27 successfully enriched samples from GCS1 and MCS3-T2 with a total of 872 774 reads across the *Bacteria*, *Archaea*, and *Eukaryota* domains, of which 613 853 were assigned to *Eukaryota* (70%). A total of 32 075 of the eukaryote reads were assigned to *Dinophyceae* (i.e. 3.68% of all domains, and 5.23% of all eukaryotes), a 61- and 15-fold increase in *Dinophyceae* reads relative to shotgun data, demonstrating the efficacy of target enrichment achieved with HABbaits1.

**Figure 4:**
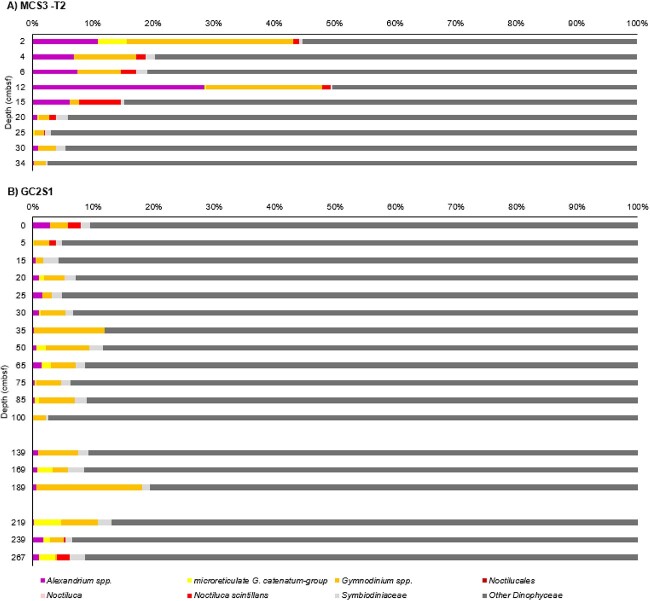
Relative *Dinophyceae* abundance (HABbaits1). *Dinophyceae* detected in non-rarefied HABbaits1 data at Maria Island coring sites (A) MCS3-T2 and (B) GC2S1, with target taxa highlighted. Highlighted are *Alexandrium* spp., the microreticulate gymnodiniods and broader genus *Gymnodinium* spp., and *N. scintillans* within the broader group Noctilucales/*Noctiluca* (total of 1 and 2 reads only, respectively). All other *Dinophyceae* are summarised, with *Symbiodiniaceae* shown separately due to potential misassignments with *Gymnodinium* or *Noctiluca* (see text). Total number of reads (both sites): 32075.

**Figure 5:**
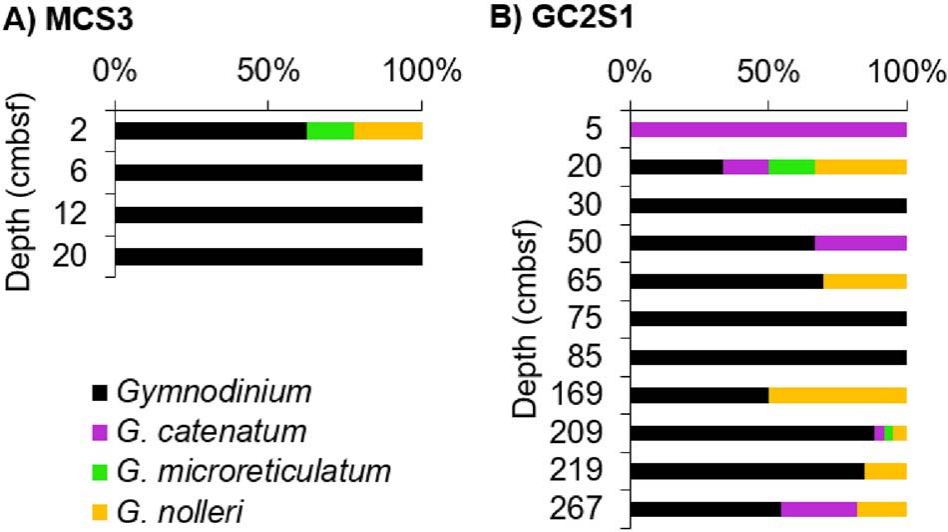
Microreticulate *Gymnodinium* species. Re-assignment of 352 *Gymnodinium* spp. at Site MCS3 (A) and GC2S1 (B) using an in-house *Gymnodinium* spp. database supplemented with additional microreticulate rRNA gene sequences. A total of 216 reads were assigned to genus and/or species level. The deepest sample at GC2S1 (267 cmbsf) should be interpreted with caution due to potential seawater contamination during core retrieval.

Hybridisation capture identified *N. scintillans*, primarily in the upper section of MCS3-T2 (above 15 cmbsf), reaching maximum relative abundance (based on 59 sequences) at 6 and 12 cmbsf (~7 and ~15 years ago, i.e. around 2010 and 2002) (Fig. 4A). *N. scintillans* was also detected in the two top samples at GC2S1 (≤8 reads each, Fig. 4B). Sequences of *Alexandrium* were most abundant at MCS3-T2, with maximum reads (854 sequences) found at 12 cmbsf (~190 years ago) from inshore core MCS3-T2 (Fig. 4A). Offshore at GC2S1, *Alexandrium* spp. were detected throughout the core at low abundance (<20 reads per sample) (Fig. 4B). A total of 352 reads were assigned to the microreticulate *G. catenatum *group at both MCS3-T2 and GC2S1, with a relatively high abundance (85 reads) inshore in MCS3-T2 2 cm (~2015). At GC2S1, microreticulate *G. catenatum*–group reads were sporadic and low abundance (≤22 reads) in the upper section (above 75 cmbsf; ~3500 years ago), and slightly more abundant (≤123 reads) below 169 cmbsf, ~6500 years ago (Fig. 4B). Verification of these 352 *G. catenatum* reads to the new *Gymnodinium* database provided 216 reads assigned to *Gymnodinium* spp. (all others remained unassigned). Most reads were assigned on genus level (165 reads) with 28, 14, and 9 assigned to *G. nolleri*, *G. microreticulatum*, and *G. catenatum*, respectively (Fig. 5). Both *G. microreticulatum* and *G. catenatum* reads were primarily found in surface sediments at MCS3 and/or GC2S1 but were also sporadically in deeper samples at GC2S1 (e.g. 209 cmbsf, ~7638 years ago). Reads assigned to *G. nolleri* were identified sporadically throughout GCS1. (Fig. 5).

While all three target dinoflagellate taxa were detected at low abundance in the deepest sample (Fig. 4B) we cannot rule out the possibility of seawater contamination of the bottom core sample during core retrieval. Dummy sample validation also indicates that shorter *Gymnodinium* and *Noctiluca* sequences may be misassigned to *Symbiodinium* (see Supplementary Material Note 3), increasing relative abundance of these taxa by ~1% (Fig. 4).

### *sed*aDNA damage analysis and authentication

**Figure 6:**
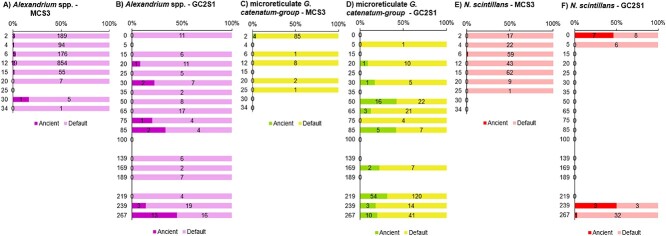
**Ancient and default reads identified for three target HAB taxa in MCS3-T2 and GC2S1.** Shown are the proportion of *sed*aDNA reads classified by HOPS analysis as ancient and default (% DNA damage) per sample based on the HABbaits1 capture sequences. The number of reads underlying these proportions are indicated within the bars.

Application of the HOPS DNA damage analysis to HABbaits1 data at site MCS3-T2 identified 30, 4, and 1 reads with ancient characteristics (signs of DNA damage) for *Alexandrium*, *G. catenatum*, and *N. scintillans*, respectively (Fig. 6, Table 1). At site GC2S1, 22, 95, and 11 “ancient” reads were identified for *Alexandrium*, *G. catenatum*, and *N. scintillans*, respectively (Fig. 6, Table 1). A relatively high number of reads passed the default filtering criteria in HOPS (Table 1), and 2% and 15% of the *Alexandrium* reads were classified as ancient in MCS3-T2 and GC2S1, respectively (Fig. 6). For microreticulate species, 4% and 27% of reads were classified as ancient in MCS3-T2 and GC2S1, respectively. For *N. scintillans*, 0.5% and 18% were classified as ancient (Table 1, Fig. 6). Target dinoflagellate reads in older sediments at offshore GC2S1 showed much more *sed*aDNA damage than younger inshore sediments at MCS3-T2.

**Table 1:** *sed*aDNA damage of reads assigned to *Alexandrium* genus, *Gymnodinium catenatum*-group and *N. scintillans*.

**Total**	**MCS3**		**GC2S1**	
**Taxa**	**Ancient**	**Default**	**Ancient**	**Default**
*Alexandrium* spp.	30	1381	22	129
*microreticulate G. catenatum*-group	4	97	95	252
*N. scintillans*	1	213	11	49
**Proportion (%)**	**MCS3**		**GC2S1**	
**Taxa**	**Ancient**	**Default**	**Ancient**	**Default**
*Alexandrium* spp.	*2*	98	*15*	85
*microreticulate G. catenatum*-group	*4*	96	*27*	73
*N. scintillans*	*0.5*	99.5	*18*	82

The total number and proportion of reads classified into ancient and default via HOPS *sed*aDNA damage analysis per site (based on HABbaits1). The proportion of ancient reads is a measure of “% *sed*aDNA damage” for each of the three species (in italics).

## Discussion

Our analysis shows that ancient dinoflagellate DNA from the genera *Alexandrium* and *Gymnodinium* is preserved in marine sediment for thousands of years. In contrast, *N. scintillans* DNA was detected primarily in recent sediments. An up to 60-fold enrichment of dinoflagellate sequences achieved by hybridisation capture with HABbaits1 arrays demonstrated the value of this approach for HAB-focused *sed*aDNA studies. Additional optimisation of hybridisation temperature and time [40, 41], with increased sequencing depth, will make it possible to maximise the ancient DNA yield of individual marine species over millennial timescales.

### Considerations for interpretation of *Alexandrium*, *Gymnodinium*, and *Noctilucased*aDNA detection

Due to the short length of ancient DNA reads (mean ~56 bp; shotgun data post-filtering), we expected a degree of uncertainty in assignment to reference sequences at a species level. However, our dummy marker gene fragment dataset runs demonstrated the value and importance of validating species assignment on a taxon-by-taxon basis.

For *Alexandrium*, the dummy sample analyses indicated that short sequences could not be confidently assigned to species, resulting in a more appropriate genus-level interpretation. In Tasmanian waters, this genus includes *Alexandrium. affine*, *A. australiense*, *A. catenella*, *A. margalefi*, *A. ostenfeldii*, *A. pacificum*, and *A. pseudogonyaulax* [1, 42, 43]. Further optimisation of RNA bait design and capture and/or gene assembly to generate longer DNA fragments may resolve this complex at the species or even regional genotype level to provide valuable information on the history and distribution of the toxic members of this genus.

The uniquely monospecific nature of the *Noctiluca* genus [44] allowed more confident species level assignments for *N. scintillans*. The few mis-assignments noted were 18S rRNA sequences (Supplementary Material Note 3, Supplementary Material Fig. 3), reinforcing conclusions that this gene is too conserved for confident species-level assignment on the basis of the short dinoflagellate reads typical of *sed*aDNA. While the HABbaits1 array included multiple markers for Tasmanian HAB dinoflagellates, baits focused on LSU-rRNA and ITS proved here to be more effective and provide higher confidence in species-level assignment of *sed*aDNA from this region and other locations [27].

While high-confidence species assignment was possible for the five microreticulate gymnodinoids, resolution was partly dependent on both fragment length and gene region/location of *sed*aDNA reads. The net effect is that only a third of microreticulate reads could be unambiguously assigned, but the reads that were assigned at the species level using the *Gymnodinium*-only database are robust. Our use of back-mapping of the ancient reads to reference alignments particularly highlights the potential for assignment artefacts arising from limited database coverage. Robust species assignments to *G. catenatum* were possible only after addition of new D3–D10 LSU-rRNA reference sequences, which also allowed detection of the non-toxic species *G. microreticulatum* and *G. nolleri*. The former is uncommon but widely distributed in both Australian and Tasmanian waters [45, 46], but the latter has not been previously identified from the Australasian region by microscopy. While *G. nolleri* appears confined primarily to coastal European waters [7], this species may be distributed widely but be rare/cryptic in Tasmanian waters.

Surprisingly, we observed very few exact sequence matches to *G. catenatum* reference sequence DQ785882 despite being in regions with sufficient variation to distinguish it from the most closely related *G. nolleri* and *G. microreticulatum*. Reference sequence DQ785882 was derived from a Korean *G. catenatum* isolate, therefore, the observed differences may partly be attributed to global variations in rRNA sequence of *G. catenatum* (e.g. [1]). Furthermore, dinoflagellate genomes possess many thousands to millions of rRNA gene loci [47], considerable intra-genomic sequence heterogeneity [48, 49], multiple sequence classes and the presence of pseudogenes [50]. As a result, the single nucleotide variation we noted among *sed*aDNA reads perhaps stems from the *sed*aDNA pipeline faithfully retaining mutations present at different rRNA loci in the original genomes.

Analysing DNA damage as a means to assess the authenticity of the ancient sequences recovered for the three target taxa, showed that the percentage of *sed*aDNA damage was low (≤4%) at the inshore site MCS3-T2. This is consistent with the earlier studies showing that eukaryote *sed*aDNA damage is very low in the upper ~35 cm of sediment at this site [30]. At GC2S1 (longer offshore core), the increased *sed*aDNA damage observed (up to 27% for *G. catenatum*) indicate sequences recovered from *Alexandrium*, microreticulate gymnodinoids and *N. scintillans* are consistent with authentic *seda*DNA, except for bottom core samples potentially contaminated by seawater during core retrieval.

### Alexandrium

The distribution of *Alexandrium sed*aDNA reads throughout the offshore core indicates that *Alexandrium* has been present in the area during the past ~9000 years. The HABbaits1 data indicate relative abundance has remained low offshore (<3%) but show a recent increase in relative abundance in inshore waters from ~15 years ago before sampling (circa 2003). The patterns of abundance observed in *sed*aDNA mirror trends observed in microscopy-based counts of *Alexandrium* resting cysts from the same cores (high/low *Alexandrium* cyst concentrations in the top 45 cmbsf at MCS3-T2/throughout GC2S1 [51]), with the trend of increased *sed*aDNA damage with depth (especially at GC2S1) supporting the authenticity of the ancient reads.

*Alexandrium* species can be prolific producers of resting cysts (approximately 40% of vegetative cells [52]). Live resting cysts are resistant to chemical and biological attack, protecting DNA from degradation during transport to the bottom sediment. Some cysts can remain dormant but viable in anoxic sediments for >100 years [53], indicating functional and undegraded DNA survival for centuries. The relative abundance patterns in our data support the view that toxic *Alexandrium* species (*A. catenella* and *A. pacificum*) are endemic but cryptic low-abundance species not previously distinguished from morphologically identical *A. australiense*. The recent increase in their relative abundance in inshore core samples also supports the view that toxic blooms detected from 2012 onwards have been stimulated by climate-induced changes in environmental conditions [10].

### *Gymnodinium catenatum* and microreticulate species

The HABbaits1 array detected microreticulate gymnodinoid sequences, including *G. catenatum*, from the most recent sediments (MCS1 2 cmbsf, year ~2015), in both inshore and offshore cores, but our refined and verified species assignments detected *G. catenatum sed*aDNA sporadically through offshore core GC2S1 (5, 20, 50, and 209 cmbsf, corresponding to ~100, ~700, ~2300, and ~7638 years ago). When sampled in 1987, *G. catenatum* represented 1% of total dinoflagellate cysts in surface sediments of Spring Bay, close to the inshore core site MCS3 [45]. However, no *G. catenatum sed*aDNA was detected at MCS3-T2 15 cmbsf (~30 years ago). It is possible that our sampling intervals missed this sediment layer.

The *sed*aDNA evidence extends the likely presence of this species well beyond the proposed 1970s introduction derived from cyst abundance in dated cores from the neighbouring Huon River [2]. Instead, our data indicates *G. catenatum* is more likely endemic but previously cryptic. While rDNA-ITS sequence polymorphisms indicate a link between Australasian populations and the Seto Inland Sea in Japan [1], our findings favour proposed alternative hypotheses: either (i) that recent dispersal between these areas could equally have been from Australasia to Japan, or (ii) that the rDNA polymorphisms are part of a wider global biogeographical pattern of natural dispersal over many thousands of years [1]. While the number of verified *G. catenatum* reads (9) was very low, the unambiguous identification highlights the known problem of overlooking low-abundance cryptic species or rare cyst types when traditional microscopy is used for detection. A companion palynological survey of the same sediment samples recorded only 3 microreticulate cysts among 4273 dinocysts counted, all were confined to the surface samples from 0 to 30 cmbsf of MCS3-T2 [51].

### Noctiluca scintillans

The HABbaits1 approach confirmed the presence of *N. scintillans* inshore of Maria Island over the last ~30 years, and traces of this dinoflagellate in recently deposited sediments offshore (surface and 5 cmbsf; <100 years old). The highest inshore relative abundances of *N. scintillans* were in the most recent sediments (from 2010 onwards; 6 and 12 cmbsf), indicating blooms occurred within this timeframe. This is consistent with previous plankton records indicating that *N. scintillans* was first detected in Tasmania in 1994 and that blooms have increased in both frequency and intensity from 2010 [11].

To our knowledge, the study reported here is the first to authenticate the presence of *N. scintillans sed*aDNA in sediment from a coastal ecosystem, demonstrating the sensitivity of the HABbaits1 approach for detecting plankton community change of fragile non-fossilising species from the marine sediment record. The absence of *N. scintillans sed*aDNA from older sediments (>100 years) offshore indicates that DNA of this soft-bodied dinoflagellate likely does not preserve well in marine sediments and/or may be considerably degraded during descent through the 104-m water column (Supplementary Material Note 6, Supplementary Material Fig. 6). Future *sed*aDNA investigations could target sediment cores from inshore coastal New South Wales around Sydney, where this species has been present for much longer. The application of similar methods to the putative range expansion of green *Noctiluca* (with green algal symbionts) into the Arabian Sea [44] would also be of considerable interest.

## Supplementary Material

Armbrechtetal_3HABS_Suppl_20240531_ycae098

## Data Availability

The demultiplexed raw sequencing data (shotgun and hybridisation capture with HABBaits1) analysed during this study are publicly available via the NCBI Sequence Read Archive (SRA) under BioProject ID: PRJNA1133055 (“Maria Island (Tasmania, Australia) *sed*aDNA, Jun 17 ‘24”; BioSample accession numbers SAMN42375555–SAMN42375603).
